# Role of Feline Coronavirus as Contributor to Diarrhea in Cats from Breeding Catteries

**DOI:** 10.3390/v14050858

**Published:** 2022-04-21

**Authors:** Sandra Felten, Ute Klein-Richers, Stefan Unterer, Michèle Bergmann, Christian M. Leutenegger, Nikola Pantchev, Jörg Balzer, Yury Zablotski, Regina Hofmann-Lehmann, Katrin Hartmann

**Affiliations:** 1 Clinic of Small Animal Medicine, Centre for Clinical Veterinary Medicine, LMU Munich, Veterinärstraße 13, 80539 Munich, Germany; u.klein@medizinische-kleintierklinik.de (U.K.-R.); s.unterer@medizinische-kleintierklinik.de (S.U.); n.bergmann@medizinische-kleintierklinik.de (M.B.); y.zablotski@med.vetmed.uni-muenchen.de (Y.Z.); hartmann@lmu.de (K.H.); 2 IDEXX Laboratories, Inc., 2825 KOVR Dr., West Sacramento, CA 95605, USA; christian.leutenegger@antechmail.com; 3 IDEXX Laboratories, Humboldtstr. 2, 70806 Kornwestheim, Germany; nikola-pantchev@idexx.com (N.P.); joerg-balzer@idexx.com (J.B.); 4 Clinical Laboratory, Department of Clinical Diagnostics and Services, Center for Clinical Studies, Vetsuisse Faculty, University of Zurich, Winterthurerstrasse 260, 8057 Zurich, Switzerland; rhofmann@vetclinics.uzh.ch

**Keywords:** endoparasites, enteritis, enteropathogen, feces, FCoV, feline enteric coronavirus, FECV, feline infectious peritonitis, FIP, RT-PCR

## Abstract

(1) Background: Feline coronavirus infection (FCoV) is common in multi-cat environments. A role of FCoV in causing diarrhea is often assumed, but has not been proven. The aim of this study was to evaluate an association of FCoV infection with diarrhea in multi-cat environments. (2) Methods: The study included 234 cats from 37 catteries. Fecal samples were analyzed for FCoV RNA by reverse transcriptase quantitative polymerase chain reaction (RT-qPCR). Potential co-infections were determined by applying a qPCR panel on different potential enteropathogens and fecal flotation. A fecal scoring system was used to categorize feces as diarrheic or non-diarrheic. (3) Results: Of the 234 cats included, 23 had diarrhea. The prevalence of FCoV infection was 87.0% in cats with and 58.8% in cats without diarrhea. FCoV infection was significantly associated with diarrhea (Odds Ratio (OR) 5.01; *p* = 0.008). In addition, presence of *Clostridium perfringens* α toxin (OR 6.93; *p* = 0.032) and feline panleukopenia virus (OR 13.74; *p* = 0.004) were associated with an increased risk of diarrhea. There was no correlation between FCoV load and fecal score. FCoV-positive cats with co-infections were not more likely to have diarrhea than FCoV-positive cats without co-infections (*p* = 0.455). (4) Conclusions: FCoV infection is common in cats from catteries and can be associated with diarrhea.

## 1. Introduction

Feline coronavirus (FCoV) is a single-stranded enveloped RNA virus belonging to the genus *Alphacoronavirus* within the family *Coronaviridae*. The virus is highly contagious and easily spreads among cats either directly via the fecal-oral route or indirectly through fomites [1]. FCoV infection is especially prevalent in crowded environments such as catteries and shelters, but also private homes with more than five cats [2,3,4]. According to the internal mutation hypothesis, FCoV exists as two distinct pathotypes [5,6]. After fecal-oral infection of a cat, FCoV replicates within enterocytes, primarily within the small intestine [7], and is shed with feces [1]. While some cats will eventually eliminate the infection and cease shedding, others can become chronic virus carriers and persistently shed FCoV in their feces [1,8,9]. In approximately 7–14% of the FCoV-infected cats in multi-cat households, however, the virus mutates within the infected cat to a highly virulent pathotype of FCoV [10], which subsequently can efficiently enter macrophages, replicate within these target cells and cause the severe disease feline infectious peritonitis (FIP) [11]. The exact nature of the mutation crucial for the change in viral cell tropism is not known. Currently, the FCoV spike gene is thought to play a central role, since its product is responsible for viral entry into macrophages [12,13,14,15,16]. Additionally, mutations within the FCoV 3c gene, a non-structural gene encoding accessory protein 3c, have been proposed to be involved in the conversion between the two FCoV pathotypes [14,17,18,19,20]. Once FCoV has developed into its highly pathogenic mutant, it is not only spread systemically within macrophages, but also leads to activation of these immune cells. Consequently, activated macrophages produce a variety of pro-inflammatory cytokines, including tumor necrosis factor alpha (TNF-α), interleukin 1β and 6, and adhesion molecules, which ultimately create a granulomatous vasculitis—the hallmark of FIP [21,22,23].

While the clinical syndrome of FIP has been extensively studied and described, little is known about the clinical consequences of natural infection with the non-mutated non-pathogenic pathotype of FCoV. Experimental studies have demonstrated the development of mild to severe enteritis after oral infection of specific pathogen-free kittens with FCoV [1,24]. Reports of natural FCoV infection associated with enteritis and diarrhea, however, are mainly limited to single case reports [25,26,27] or case series [28]. Additionally, although some more recent studies have shown that prevalence of fecal FCoV shedding can be high in cats with diarrhea [29,30], it is not known whether the diarrhea is a direct consequence of FCoV infection. Co-infections with other enteropathogens are common in cats with FCoV infection, especially in those in multi-cat environments [31,32] and the impact of these and other factors, such as drugs or systemic diseases, on the occurrence of gastrointestinal disease has not been sufficiently considered in most studies. Thus, a direct link between FCoV infection and the development of diarrhea has not been proven under field conditions.

Therefore, it was the aim of this study to evaluate the role of FCoV as a pathogen potentially contributing to diarrhea in cats from multi-cat households in the field, and to determine a possible correlation of the amount of FCoV shedding and the severity of diarrhea. Additionally, the presence of other infectious agents potentially causing diarrhea was investigated.

## 2. Materials and Methods

### 2.1. Cats

This prospective study included catteries that kept at least five cats and at least one intact queen for breeding. In order to eliminate confounding factors, five cats were excluded from the study because they had a known history of diseases or had received treatments that can potentially cause diarrhea. These conditions included surgical resection of a large portion of the small intestine (*n* = 1), suspected food intolerance (*n* = 2), hyperthyroidism (*n* = 1), and megacolon (*n* = 1). The final study population consisted of 234 cats from 37 catteries. Breeds included British Shorthair (*n* = 57), Bengal (*n* = 51), Birman (*n* = 32), Norwegian Forest Cats (*n* = 22), Persian (*n* = 18), Maine Coon (*n* = 17), Somali (*n* = 8), Turkish Van (*n* = 7), Scottish Fold (*n* = 4), Scottish Straight (*n* = 4), Sphynx (*n* = 4), Savannah (*n* = 3), Turkish Angora (*n* = 3), Oriental (*n* = 1), and Taiga (*n* = 1). The breed of two cats was not recorded. Of the 234 cats, 79 were male (65 sexually intact, 14 neutered) and 155 were female (137 sexually intact, 18 neutered). The age of the cats ranged from 2 months to 12 years. Fifty-three cats were <1 year of age, 147 cats were 1–5 years of age, and 32 cats were >5 years of age. The age of two cats was not recorded. Of the 37 catteries, 16 housed 5–10 cats and 21 housed > 10 cats. Breeders were free to sample as many cats as desired. As a consequence, not all cats from each cattery were included in the study.

### 2.2. Samples

Samples were collected from February 2016 to September 2017. One fecal sample was collected from each cat and analyzed within 48 h of defecation. Fecal samples were kept at 4 °C until analysis. Fecal consistency of each sample was determined always by the same investigator (U.K.-R.) using a visual scoring chart with a score ranging from 1 to 7 (Purina fecal score, Nestlé Purina, St. Louis, MO, USA; Table 1). For statistical analysis, diarrhea was defined as abnormal fecal consistency and fecal samples were divided into two categories: normal fecal consistency (scores 1–3) or diarrheic feces (scores 4–7) based on the visual fecal score. Defecation frequency could not be assessed, since only one fecal sample was analyzed per cat.

### 2.3. Detection of FCoV

**Figure 1:**
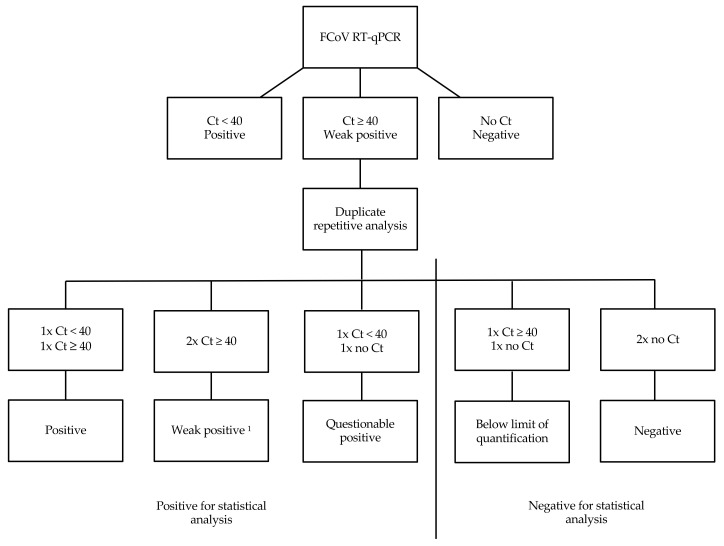
Interpretation of results of the reverse transcriptase quantitative polymerase chain reaction (RT-qPCR) for feline coronavirus (FCoV) RNA. Ct = threshold cycle; ^1^ Low concentration of FCoV RNA detected.

All fecal samples were examined for FCoV RNA load per gram (g) of feces by reverse transcriptase quantitative polymerase chain reaction (RT-qPCR) as described previously [33]. For this, total nucleic acid was extracted from fecal samples applying the “MagVet™ Universal Kit” (ThermoFisher, Darmstadt, Germany) on a KingFisher Flex Purification System platform (ThermoFisher, Darmstadt, Germany) according to the manufacturer instructions. RT-qPCR based on the FCoV 7b gene [33] was performed as a singleplex reaction. RT-qPCR was run with six quality controls, including RT-qPCR-positive controls, RT-qPCR-negative controls, negative extraction controls, an internal positive control (IPC) spiked into the lysis solution to monitor the nucleic acid extraction efficiency and presence or absence of inhibitory substances, RNA quality control, and an environmental contamination monitoring control [31]. Samples with a threshold cycle (Ct) value below 40 were considered positive. If RT-qPCR was initially weak positive (Ct ≥ 40), RT-qPCR was repeated in duplicate. Depending on the results of this duplicate repetitive analysis, results were then categorized (Figure 1).

### 2.4. Detection of Other Potential Enteropathogens

Additionally, all fecal samples were examined by quantitative PCR and fecal flotation for a panel of other potential enteropathogens [30]. Target genes for enteropathogen detection using real-time PCR are shown in Table 2.

Quality controls were used as stated above. Quantification of *C. perfringens* toxin gene DNA was performed as described before [34]. Cats were considered positive for *C. perfringens* encoding for α toxin (*cpa*) if the copy number was >300,000 per g of feces.

Fecal flotation to determine *Giardia* spp., coccidia, *Taenia*-type eggs (*Taenia/Echinococcus*) and *Ancylostoma tubaeforme* was performed using a dilution of 1 g of feces and 15 mL sugar solution (specific gravity 1.27, as determined by aerometer). The suspension was strained into a tube through a gauze pad and centrifuged at 350 G for 10 min with a cover slip on the meniscus. The cover slip was then removed and placed on a microscopic slide for examination at 400× magnification.

### 2.5. Statistical Analysis

Data analysis was performed using R version 4.0.3 (10 October 2020, R Foundation for Statistical Computing, Vienna, Austria). Results with a *p*-value < 0.05 were considered statistically significant. Proportions of diarrheic vs. non-diarrheic animals in every enteropathogen group were compared with a 2-sample test for equality of proportions with Yates’ continuity correction. Yates’ continuity correction was applied due to small sample sizes. The influence of every potential enteropathogen on diarrhea (fecal score ≥ 4) was first studied by univariate analysis, as recommended by Dohoo et al. [35], using two different methods, logistic regression and Chi-Square test with simulated *p*-values. The *p*-values were simulated due to small sample sizes. The exact 95% confidence intervals (95% CI) for percentages were calculated using Clopper–Pearson method. In all enteropathogens, in which the *p*-value of the univariate analysis was ≤0.1, the enteropathogen was suspected to have an association with diarrhea and was further analyzed in the multiple logistic regression.

In order to exclude young age as confounding factor, a Mann–Whitney U test was applied to compare the likelihood of diarrhea between younger and older cats. Additionally, a Mann–Whitney U test was performed to compare the likelihood of being infected with enteropathogens significantly associated with diarrhea between younger and older cats.

The difference in fecal scores and FCoV loads between FCoV-positive cats with and without additional potential enteropathogens was evaluated via the Mann–Whitney U test.

## 3. Results

The feces of 23 of the 234 cats were considered diarrheic (fecal score ≥ 4): 17 cats had a fecal score of 4, four cats had a fecal score of 5, and two cats had a fecal score of 6. None of the cats had a fecal score of 7 (Table 3). All cats were considered clinically healthy by their owners except for the altered fecal consistency. Prevalence of FCoV infection among diarrheic cats was 87.0% (20/23 cats), which was significantly higher than the prevalence of 58.8% (124/211 cats) in non-diarrheic cats (*p* = 0.016). All of the 23 diarrheic cats and 200/211 non-diarrheic cats were positive for at least one fecal pathogen. 

In 144 of the 234 cats (61.5%), FCoV RNA was detected in feces by RT-qPCR (Table 3). The proportion of cats positive for FCoV RNA within the individual catteries ranged from 10% to 100% of the sampled cats. In five catteries, all sampled cats were negative for FCoV RNA. Mean fecal FCoV load was 1.05 × 10^11^ viral copies per g of feces (range 1.98 × 10^6^–1.71 × 10^12^). Four cats had a weak positive FCoV RT-qPCR result with an initial Ct value > 40. In these four cats, duplicate repetitive analysis was performed. After this duplicate analysis, one cat was defined as weak positive, two cats questionable positive, and one cat was positive, but the FCoV load was below the limit of quantification. Weak positive and questionable positive were considered positive for statistical analysis (Figure 1), but were excluded from analysis of correlation between fecal score and fecal FCoV load, since fecal FCoV load could not be determined. The sample that was below the limit of quantification was considered negative for FCoV for statistical analysis. The results for FCoV (as well as the panel of other potential enteropathogens) by different fecal scores are shown in Table 3 and Table 4. 

Five potential enteropathogens (FPV, FCoV, *cpa*, *Ancylostoma tubaeforme*, *Salmonella enterica*) were shown to have a potential association with diarrhea in both univariate analyses. *Ancylostoma tubaeforme* and *Salmonella enterica* showed the weakest association in univariate analyses (both *p* = 0.17) and did not show a significant association in the multivariate model (both *p* ≥ 0.17). Infection with FCoV, *cpa*, and FPV was significantly associated with an increased risk of having diarrheic feces in the multiple logistic regression (Table 4).

There was a tendency for young cats to have diarrhea more frequently than older cats. However, this difference was not significant (median age of cats with diarrhea 16 months vs. 23 months in cats without diarrhea; *p* = 0.07). Additionally, young cats tended to be infected with FPV more frequently, but again, this was not statistically significant (median age in FPV-infected cats 16.5 months vs. 23 months in non-infected cats, *p* = 0.08). Infection with FCoV (median age in FCoV-infected cats 22 months vs. 23 months in non-infected cats, *p* = 0.60) or *cpa* (median age in *cpa*-infected cats 22 months vs. 27 months in non-infected cats, *p* = 0.16) was not significantly more common in young cats compared to older cats.

**Figure 2:**
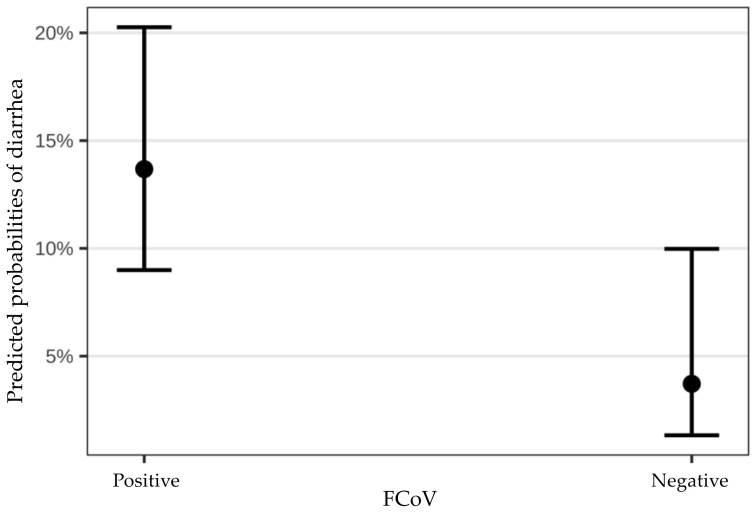
Bootstraps demonstrating the comparison of the risk of diarrhea in cats positive vs. cats negative for feline coronavirus (FCoV) RNA. Black points represent the mean, 95% confidence intervals are shown.

When comparing FCoV-positive and FCoV-negative cats, FCoV-positive cats had a significantly higher risk of having a fecal score ≥4 (*p* = 0.015, Odds Ratio (OR) 4.10, 95% confidence interval 1.54–20.49) (Figure 2).

**Figure 3:**
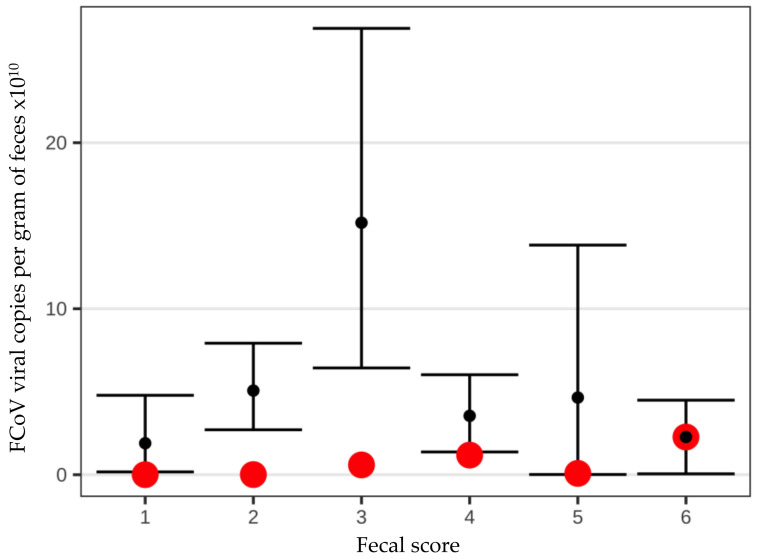
Bootstraps demonstrating no correlation between fecal score and fecal feline coronavirus (FCoV) load in viral copies per gram (g) of feces. Black points represent the mean fecal FCoV load in viral copies per g of feces. The 95% confidence intervals for the mean were calculated via the basic nonparametric bootstrap without assuming normality. Red points represent the median fecal FCoV load in viral copies per g of feces.

However, there was no correlation between FCoV virus load per g of feces and fecal score (Figure 3).

**Figure 4:**
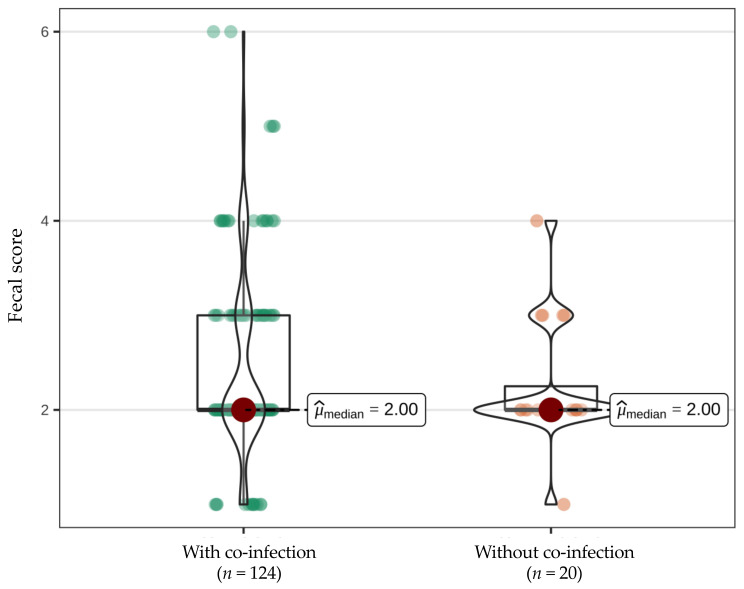
Box-Violin plots with medians (red points) display the non-normally distributed nature of the fecal score and demonstrate that there is no difference in fecal score between cats with feline coronavirus (FCoV) infection with or without co-infection with other potential enteropathogens (*p* = 0.455) according to the nonparametric Mann–Whitney U test.

**Figure 5:**
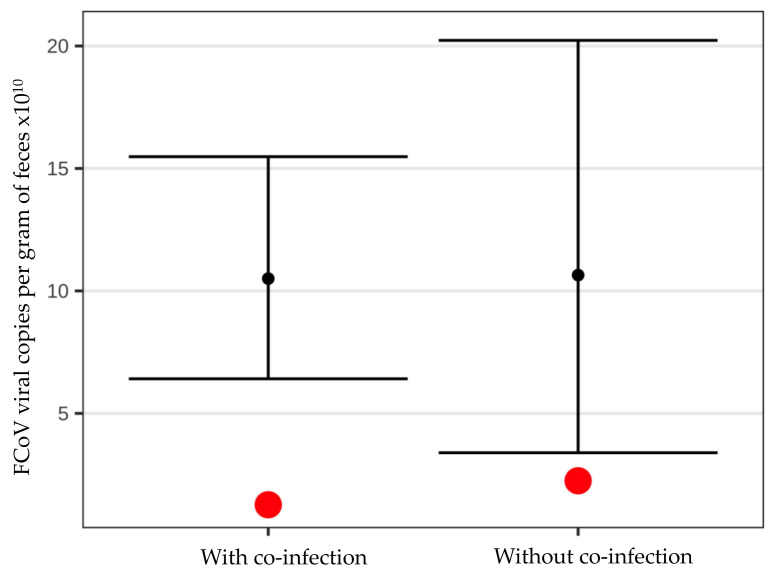
Bootstraps demonstrating the difference in fecal feline coronavirus (FCoV) load in viral copies per gram (g) of feces between FCoV-infected cats with and without co-infection with other potential enteropathogens. Black points represent the mean fecal FCoV load in viral copies per g of feces. Red points represent the median fecal FCoV load in viral copies per g of feces.

Additionally, most of the FCoV-infected cats (124/144, 86.1%) had at least one co-infection with another potential enteropathogen. Of the 20 FCoV-positive cats with diarrhea, 19 (95.0%) had at least one co-infection with another potential enteropathogen. Of the 124 FCoV-positive cats without diarrhea, 103 (83.1%) had at least one co-infection with another potential enteropathogen. Nevertheless, there was no statistically significant difference in median fecal score (*p* = 0.455; Figure 4) or median fecal FCoV load (*p* = 0.423; Figure 5) between FCoV-positive cats with and without additional potential enteropathogens.

## 4. Discussion

Prevalence of FCoV shedding in the feces of cats from German catteries in this study was 61.5%. Previously described FCoV shedding prevalences vary greatly, depending on the geographic location and housing conditions of the cats. Reported prevalences range from 32.7% to 56.9% [3,31,32]. The somewhat higher prevalence noted in the present study can be explained by the inclusion of only cats from catteries housing at least five cats and the fact that FCoV infection is especially prevalent in multi-cat households [2,4,36]. Only one fecal sample was examined from each cat in this study. Cats infected with FCoV can shed the virus intermittently [9], and it has been shown earlier that at least three fecal samples should be collected for FCoV RT-qPCR from each cat in order to correctly identify FCoV shedders [2]. Therefore, FCoV shedding prevalence likely would have been higher in the present study if serial fecal samples had been analyzed, and it cannot be excluded that some cats shedding FCoV intermittently were missed.

While studies on FIP are numerous, studies on the clinical course of infection with non-mutated FCoV are rather sparse and most often are of experimental design [1,37]. It has been stated before that natural FCoV infection can cause diarrhea and even fatal enteritis in cats, especially kittens, and that diarrhea was significantly associated with FCoV antibody-positivity [28,38]. However, none of these previous studies evaluated confounding factors such as other potential enteropathogens, and thus, a causative link between FCoV infection and the occurrence of diarrhea in cats has never been clearly established in cats with naturally occuring FCoV infection. The results of the present study indicate that FCoV infection indeed is significantly associated with diarrhea. The prevalence of FCoV shedding among cats with diarrheic feces was 87.0%; in contrary, it was only 58.8% in cats with normal fecal consistency. When comparing FCoV-infected and FCoV-non-infected cats, FCoV-infected cats had a four times higher risk of having diarrhea than FCoV-negative cats. These results are comparable to those of a study evaluating enteric infections in cats from hoarding environments reporting a prevalence of FCoV infection of 88% in cats with diarrhea [30]. However, the prevalences and odds in the present study were even higher than those reported in other previous studies. In a study investigating the occurrence of potential enteropathogens in cats in Florida, USA, FCoV prevalence among cats with diarrhea was 58% compared to a prevalence of 36% in cats with normal feces. The odds of cats with diarrhea being FCoV-infected in that study was 2.46 times that of cats without diarrhea [32]. A similar prevalence of FCoV shedding of 33% was confirmed in healthy cats from California, USA [3]. The discrepancy between those results and the results of the present study can be explained by the cat population included in the studies. Both former studies examined fecal samples from cats that were just entering an animal shelter, meaning that they did not live under crowded conditions before sampling. It could be demonstrated that FCoV shedding prevalence increased to 60% within one week of being in a shelter environment [3]. Cats in the present study, in contrast, were already living in a multi-cat environment, generally for their entire lives, which can explain the higher shedding prevalence. In a study describing the course of natural FCoV infection among kittens in catteries, kittens with diarrhea had a 2.85 times increased risk of having anti-FCoV antibodies compared to kittens without diarrhea, but no fecal shedding was determined in that study [38]. 

Overall, FCoV was associated with the occurrence of diarrhea in the cats in the present field study. Given this association, it should be considered to include fecal RT-qPCR in the diagnostic plan for cats with diarrhea, especially in cats from multi-cat households. However, due to the high number of co-infections noted in the present study, additional testing for potential enteropathogens by fecal flotation and specific PCR is also mandatory. Additionally, it has to be kept in mind that, although FCoV seems to play a role in the development of diarrhea, other (potentially more common) causes for diarrhea, such as food intolerance, chronic enteropathy or endocrinopathies, need to be considered in every cat presented for gastrointestinal signs as well. This might be especially true for cats with a chronic course of disease. Standardized diagnostic investigations, such as endoscopy, for gastrointestinal signs were not performed in all of the cats of the present study and the causes of diarrhea can be quite variable in individual cats. Therefore, it cannot be excluded that increased stress levels that might be present in cats living under crowded conditions of multi-cat households promote a higher prevalence of both FCoV infection and gastrointestinal problems. Thus, detection of any potential enteropathogen in the feces of a cat with diarrhea does not inevitably indicate that this pathogen is responsible for the gastrointestinal signs. Additionally, the question of whether FCoV really is a causative agent of diarrhea in cats likely can only be answered unequivocally by performing experimental infection studies [1,24,37], which, however, do not reflect the situation in privately owned multi-cat households, which is encountered by cat breeders and veterinarians. Therefore, the aim of this study was to evaluate the role of FCoV as a potential contributor to diarrhea in a field situation. The study design used does, however, not allow to establish a causal relationship between FCoV infection and diarrhea in cats.

FCoV-infected cats shedding large amounts of viral particles with their feces pose a significant risk to other cats living in the same household [39,40]. These high-intensity shedders are also known to often shed FCoV for a prolonged period of time [41]. It could have been anticipated that increased viral replication within enterocytes leading to high fecal virus loads would also cause more extensive lesions within the gastrointestinal tract, and thus, more severe diarrhea. However, interestingly, the present study did not find a significant association between fecal FCoV load and fecal score. Nevertheless, only one fecal sample was examined from each cat, and consequently, day-to-day variations in fecal consistency and FCoV shedding quantity cannot be excluded.

In order to exclude the influence of other potential enteropathogens on fecal consistency, the fecal score of FCoV-infected cats without co-infections was compared to FCoV-infected cats with co-infections, which did not result in a significant difference.

Other enteropathogens that were significantly associated with an increased risk of diarrheic feces were *C. perfringens* encoding for α toxin gene (*cpa*) and FPV. However, the role of *C. perfringens* as enteropathogen in cats is debatable. It is possible that an increased rate of detection of *cpa* merely is a manifestation of intestinal dysbiosis. While detected in 95.7% of cats with diarrhea, the α toxin gene was also detected in the majority (79.1%) of non-diarrheic cats and confidence intervals were rather wide. In former studies, *C. perfringens* could be detected in up to 63% of healthy cats and the α toxin is present in virtually all *C. perfringens* strains and therefore is not a marker of virulence [42]. Thus, the relevance of infection with *C. perfringens* encoding for α toxin gene in the cats of the present study seems limited. 

The overall prevalence of FPV infection in the study population was 2.6% and as such comparable to a study from Florida [32]. The prevalence in cats with diarrhea was 13.0%, which is much lower than the prevalence reported in cats with diarrhea from the UK. In that study, FPV prevalence was 22.1% [31] and an even higher prevalence of 41.4% was demonstrated in diarrheic cats from Japan [43]. Nevertheless, prevalence of FPV in cats with diarrhea in the present study was still significantly higher than in non-diarrheic cats. None of the cats with FPV infection in the present study had clinical signs except for mild diarrhea (fecal score 4 or 5) in two cats. Mild infection is a likely explanation for this, likely because most of the cats were vaccinated against FPV. In a study from the UK, canine parvovirus was found in the feces of 34% healthy cats [44]. Positive PCR results due to recent vaccination with modified-live virus vaccines [45] cannot fully be excluded, since vaccination history of the cats was unknown.

This study had some limitations. First, all of the cats included were from cattery housing groups of multiple cats, leading to close contact between the individual cats and sharing of litter boxes. Each of the catteries housed at least one cat positive for FCoV or other potential enteropathogens. Therefore, cross-contamination between the fecal samples from the cats cannot fully be excluded. Nevertheless, cat breeders acted with great caution to separate fecal samples. Second, only one fecal sample was collected for analysis from each cat and the classification into diarrheic and non-diarrheic was based on the determination of fecal score from this one sample. As a consequence, variations in fecal consistency over time and defecation frequency could not be evaluated, although persistence of diarrhea is an important factor to be considered. It cannot be excluded that there was some misclassification of cats into the categories “diarrheic” and “non-diarrheic” based on fecal consistency of only one fecal sample. Fecal consistency can, for instance, be influenced by dietary or environmental factors. Thus, some cats might have had intermittent diarrhea, and therefore, it is possible that these cats were missed by examining only one fecal sample. Additionally, it is possible that some FCoV-infected cats shedding FCoV intermittently were missed, since at least three fecal samples are necessary to reliably identify FCoV shedders [2]. This could also be an explanation why in five catteries, all sampled cats were negative for FCoV RNA and indicates that this finding has to be interpreted cautiously. Further studies should include a larger number of cats with diarrhea based on the examination of at least three consecutive samples and possibly also altered frequency of defecation.

## 5. Conclusions

The results of the present study indicate that FCoV infection is associated with the occurrence of diarrhea in cats. Fecal RT-qPCR testing for FCoV therefore should be considered in cats with abnormal fecal consistency, especially if they are living in multi-cat environments. However, co-infection with various other potential enteropathogens is very common, and therefore, additional fecal testing is recommended. Additionally, since FCoV and potential enteropathogens frequently can be identified in non-diarrheic cats, the presence of specific bacterial strains, viruses, and protozoa is not per se diagnostic, and a standardized diagnostic approach to diarrhea is still warranted.

## Figures and Tables

**Table 1:** Fecal score (Purina fecal score, Nestlé Purina, St. Louis, USA) used to classify fecal samples.

Fecal Score	Fecal Consistency
1	Very hard and dry
2	Firm, but not hard, pliable
3	Log-shaped, moist surface, holds form when picked up
4	Very moist and soggy, loses form when picked up
5	Very moist, but distinct shape
6	Has texture, but no distinct shape
7	Watery, no texture

**Table 2:** Method of detection and target genes for detection of potential enteropathogens using quantitative polymerase chain reaction (qPCR) or reverse transcriptase quantitative polymerase chain reaction (RT-qPCR) and fecal flotation.

Enteropathogen	Method of Detection	Target Gene
Feline coronavirus	RT-qPCR	7b gene
Feline panleukopenia virus	qPCR	VP2 gene (EU252145)
*Clostridium perfringens* encoding for α toxin gene	qPCR	α toxin gene (AM888388)
*Clostridium perfringens* encoding for enterotoxin gene	qPCR	Enterotoxin gene (KU711834.1)
*Campylobacter jejuni/coli*	qPCR	lpxA gene (AY531523.1; AY531498.1)
*Salmonella enterica*	qPCR	Invasion A gene (EU348366)
*Cryptosporidium* spp.	qPCR	Small subunit rRNA gene (A093489)
*Tritrichomonas foetus*	qPCR	5.8S rRNA gene (AF339736)
*Giardia* spp.	qPCR and fecal flotation	Small subunit rRNA gene (DQ836339)
*Toxoplasma gondii*	qPCR and fecal flotation	Internal transcribed spacer-1 gene (L49390)
Coccidia	Fecal flotation	n. a.
*Taenia*-type (*Taenia/Echinococcus)* spp.	Fecal flotation	n. a.
*Ancylostoma tubaeforme*	Fecal flotation	n. a.

n. a. = not applicable; spp. = species.

**Table 3:** Number of cats positive for feline coronavirus (FCoV) and other potential enteropathogens grouped by fecal score.

Fecal Score	FCoV	FPV	*cpa*	*cpe*	*Campylobacter jejuni/coli*	*Salmonella enterica*	*Crypto-* *sporidium* spp.	*T. foetus*	*Giardia* spp.	*Toxoplasma gondii*	Coccidia	*Taenia*-Type (*Taenia/Echinococcus)* spp.	*Ancylostoma* *tubaeforme*
1 (*n* = 23)	13	1	20	3	2	1	0	3	2	0	0	0	0
2 (*n* = 146)	81	1	114	13	6	0	3	8	17	1	3	3	1
3 (*n* = 42)	30	1	33	2	1	0	5	2	4	0	2	0	0
4 (*n* = 17)	15	2	16	0	2	1	0	1	1	0	0	0	1
5 (*n* = 4)	3	1	4	0	0	0	1	0	0	0	0	0	0
6 (*n* = 2)	2	0	2	0	0	0	0	0	0	0	0	0	0
7 (*n* = 0)	0	0	0	0	0	0	0	0	0	0	0	0	0
**Total** **(%)**	144 (61.5)	6 (2.6)	189 (80.8)	18 (7.7)	11 (4.7)	2 (0.9)	9 (3.8)	14 (6.0)	24 (10.3)	1 (0.4)	5 (2.1)	3 (1.3)	2 (0.9)

*cpa* = *Clostridium perfringens* encoding for α toxin gene; *cpe* = *Clostridium perfringens* encoding for enterotoxin gene; FPV = feline panleukopenia virus; spp. = species; *T. foetus* = *Tritrichomonas foetus*.

**Table 4:** Prevalence (%) and 95% confidence intervals (95% CI) of feline coronavirus (FCoV) and other potential enteropathogens in the 234 cats. Cats were grouped into diarrheic feces (fecal score ≥ 4) and non-diarrheic feces (fecal score < 4). The exact 95% CI for percentages were calculated using the Clopper–Pearson method. The influence of every potential enteropathogen on diarrhea was first studied by univariate analysis. If the *p*-value of the univariate analysis was ≤0.1, the enteropathogen was included in a multiple logistic regression. *p*-values and Odds Ratios are shown for the final multivariate model.

Group	FCoV	FPV	*cpa*	*cpe*	*Campylo-* *bacter* *jejuni/coli*	*Salmonella enterica*	*Crypto-sporidium* spp.	*T. foetus*	*Giardia* spp.	*Toxoplasma gondii*	Coccidia	*Taenia*-Type (*Taenia/Echinococcus)* spp.	*Ancylostoma* *tubaeforme*
Diarrheic (*n* = 23)(95% CI)	87.0(66.4–97.2)	13.0 (2.8–33.6)	95.7 (78.1–99.9)	0.0 (0.0–1.0)	8.7 (1.1–28.0)	4.3 (0.1–21.9)	4.3 (0.1–21.9)	4.3 (0.1–21.9)	4.3 (0.1–21.9)	0.0 (0.0–1.0)	0.0 (0.0–1.0)	0.0 (0.0–1.0)	4.3 (0.1–21.9)
Non-diarrheic(*n* = 211)(95% CI)	58.8(51.8–65.5)	1.4 (0.3–4.1)	79.1 (73.0–84.4)	8.5 (5.1–13.1)	4.3 (2.0–7.9)	0.5 (0.0–2.6)	3.8 (1.7–7.3)	6.2 (3.3–10.3)	10.9 (7.0–15.9)	0.5 (0.0–2.6)	2.4 (0.8–5.4)	1.4 (0.3–4.1)	0.5 (0.0–2.6)
*p*-value	0.008	0.004	0.032	n. s.	n. s.	n. s.	n. s.	n. s.	n. s.	n. s.	n. s.	n. s.	n. s.
Odds Ratio(95% CI)	5.01 (1.51–16.60)	13.74 (2.28–82.77)	6.93 (1.17–41.05)	n. s.	n. s.	n. s.	n. s.	n. s.	n. s.	n. s.	n. s.	n. s.	n. s.

*cpa* = *Clostridium perfringens* encoding for α toxin gene; *cpe* = *Clostridium perfringens* encoding for enterotoxin gene; FPV = feline panleukopenia virus; n. s. = not significant; spp. = species; *T. foetus* = *Tritrichomonas foetus*.

## Data Availability

Not applicable.
